# A Scoping Review of Preclinical Environmental Enrichment Protocols in Models of Poststroke to Set the Foundations for Translating the Paradigm to Clinical Settings

**DOI:** 10.1007/s12975-025-01335-3

**Published:** 2025-02-06

**Authors:** Luca Oppici, Guna Bērziņa, Ann Marie Hestetun-Mandrup, Marianne Løvstad, Arve Opheim, Matheus M. Pacheco, Lena Rafsten, Katharina S. Sunnerhagen, PEER-HOMEcare consortium, James R. Rudd

**Affiliations:** 1 Department of Teacher Education and Outdoor Studies, Norwegian School of Sport Sciences, 0863 Oslo, Norway; 2 Department of Rehabilitation, Faculty of Health and Sport Sciences, Riga Stradiņš University, Riga, Latvia; 3 Clinic of Rehabilitation, Riga East University Hospital, Riga, Latvia; 4 Sunnaas Rehabilitation Hospital, 1450 Nesoddtangen, Norway; 5 Department of Rehabilitation Science and Health Technology, Oslo Metropolitan University, Oslo, Norway; 6 Department of Psychology, University of Oslo, Oslo, Norway; 7 Institute of Neuroscience and Physiology, Dept of Clinical Neuroscience and Rehabilitation Medicine, Sahlgrenska Academy, University of Gothenburg, Gothenburg, Sweden; 8 Faculty of Sport, University of Porto, CIFI2D Porto, Portugal; 9 Department of Occupational Therapy and Physiotherapy, Sahlgrenska University Hospital, Gothenburg, Sweden; 10 Department of Sport, Food and Natural Sciences, Faculty of Education, Arts and Sports, Western Norway University of Applied Sciences, 6856 Sogndal, Norway

**Keywords:** Enriched environment, Translational, Stroke rehabilitation, Neuroplasticity

## Abstract

The translation of the highly effective Environmental Enrichment (EE) paradigm from preclinical animal models to human clinical settings has been slow and showed inconsistent results. The primary translational challenge lies in defining what constitutes an EE for humans. To tackle this challenge, this study conducted a scoping review of preclinical EE protocols to explore what constitutes EE for animal models of stroke, laying the foundation for the translation of EE to human application. A systematic search was conducted in the MEDLINE, PsycINFO, and Web of Science databases to identify studies that conducted an EE intervention in the post-stroke animal model. A total of 116 studies were included in the review. A critical reflection of the characteristics of the included studies revealed that EE for post-stroke is a strategy that frequently modifies the animals’ daily environment to create a richness of spatial, structural, and/or social opportunities to engage in a variety of daily life-related motor, cognitive, and social exploratory activities. These activities are relevant to the inhabiting individual and involve the activation of the body function(s) affected by the stroke. This review also identified six principles that underpinned the EE protocols: complexity (spatial and social), variety, novelty, targeting needs, scaffolding, and integration of rehabilitation tasks. These findings can be used as steppingstones to define what constitutes EE in human clinical applications and to develop a set of principles that can inform the design of EE protocols for patients after a stroke.

**Supplementary Information:**

The online version contains supplementary material available at 10.1007/s12975-025-01335-3.

## Introduction

Environmental Enrichment (EE), also called enriched environment, refers to an experimental paradigm where the living conditions of an individual(s) are modified to increase physical and social stimulation [1]. EE has been shown to provide enhanced motor, cognitive, and sensory stimulation to animals with morbidity [2], and it represents a key paradigm for investigation to enhance post-stroke rehabilitation [3]. Evidence indicates that housing animal models of stroke in an EE improves the rehabilitation process through a series of nested mechanisms, such as neurogenesis, increased cortical thickness, and reduction of white matter damage (for a detailed review of the mechanisms, see [3, 4]). These mechanisms underly an enhancement of cognitive and motor functions [5], ultimately leading to an increased autonomy to perform daily functions. The large body of preclinical evidence has generated interest in the clinical community, sparking optimism on the potential of applying principles of EE to improve the rehabilitation after stroke [6, 7]. The translation of the EE paradigm from preclinical to clinical settings however has not yielded the same promise that has been observed in the animal models [8, 9]. The primary translational challenge lies in defining what constitutes an EE for humans [3, 7]. To tackle this challenge, this study conducts a scoping review of preclinical EE protocols to explore what constitutes EE for animal models of stroke, laying the foundation for the translation of EE to human applications.

What constitutes EE in animal models of stroke? The initial and most widely used definition of EE is a “combination of social and inanimate stimulation” [1, 10], which was then refined to “housing condition, either home cages or exploratory chambers, that facilitate enhanced sensory, cognitive and motor stimulation” [2] and “enriched environment provides the animals with optimal conditions for enhanced exploration, cognitive activity, social interaction and physical activity” [11]. These definitions converge towards EE being an environment that stimulates enhanced motor, cognitive, and exploratory activities. What constitutes a stimulating environment though has not been systematically scrutinised and defined. To create a stimulating environment, it is common understanding and practice to add elements to an impoverished cage, i.e., a bare cage with a limited number of animals. For instance, studies can increase the size of the cage, or add animal peers, objects, toys, playing objects, or structural layers. What elements are added and how they are added vary across studies.

A standardization of the EE protocol could reveal and align perspectives on what constitutes EE. However, this is hardly achievable due to the large variability across experimental conditions, animal genetics, and lab environments [12]. Also, in order to move forward in translating EE models to human conditions, it is theoretically more relevant to understand the key set of principles and approaches that underly the approaches in EE models. This will be useful in guiding individualized human interventions [13].

Researchers have put forward the principles of complexity, variability, and novelty to design EE procedures that can drive physical activity, cognitive activity (e.g., learning), sensory stimulation, and exploratory behaviour [14, 15]. Complexity in the structural and spatial layout of the cage environment [16, 17], novelty and variability in the provision of stimuli to encourage the exploration of novel and alternative solutions and provide a diversity of experiences [13]. The animals’ needs (e.g., feeding, sheltering, and doing physical activity) and preferences are suggested to be another important aspect of EE [13]. Targeting the animals’ needs can stimulate them to engage in physical and cognitive activities to satisfy those needs. For instance, placing food pellets at different levels of the cage stimulate animals to move around and explore to find food and satisfy their feeding need.

Despite previous attempts to clarify the basis of EE, to date, no study has provided a systematic and comprehensive overview of the variety of EE protocols conducted and design principles adopted in preclinical post-stroke models. It is therefore unclear what constitutes an EE and how the principles of complexity, variability, and novelty are implemented, what animals’ needs and preferences are considered when an EE intervention is conducted, and whether additional design principles are used.

The aim of this study is to examine what constitutes EE in the preclinical post-stroke literature (basic science) in order to lay a stepping stone in the translation to clinical settings. A scoping review of the EE protocols in post-stroke animal models and a critical reflection on common concepts and principles adopted was conducted. The scoping review approach was chosen to provide a full picture of all the protocols published to-date. Reviews of preclinical studies are often viewed as key initial stages in translational research [18], and the results of this review have the potential to offer valuable insights for the understanding of EE that can be explored and tested in clinical settings.

## Methods

The current study follows the guidelines proposed by the Preferred Reporting Items for Systematic reviews and Meta-Analyses extension for Scoping Reviews (PRISMA-ScR [19], see checklist in the supplementary material).

### Eligibility Criteria

Considering that this review does not aim to synthesize the effectiveness of an intervention, the eligibility criteria contain only the Population and Intervention of the PICOS statement. Studies that conducted an EE intervention with an animal post-stroke model, peer-reviewed and published in English were included in the review (see supplementary material for the full list of inclusion and exclusion criteria).

### Information Sources and Search Strategy

Three databases were searched: MEDLINE, PsycINFO, and Web of Science, from the inception of literature to June 26, 2024. Additionally, relevant review articles and the reference list of the included studies were screened to identify additional studies to include.

The search string comprised the “stroke” and “enriched environment” domains, resulting in the following syntax: (stroke OR “cerebral infarct” OR “cerebrovascular accident” OR “CVA” OR “brain ischemia” OR “focal cortical ischemia” OR “cortical infarct” OR “cerebral ischemia” OR “MCA occlusion” OR “middle cerebral artery occlusion” OR “brain hypoxia”) AND ("enriched environment" OR "environmental enrichment" OR "enriched housing"). The search string for each database is presented in the supplementary material.

### Selection Process

The records identified through the database searching were exported into Endnote X9 software (Clarivate, Philadelphia, USA). Duplicates were first removed automatically, and then the database was manually checked for unrecognized duplicates. The screening was first performed on manuscript titles, then on abstracts, and ultimately on full article texts. Two raters (author LO and a research assistant) independently screened the records, cross-checked their results, and resolved any conflict. If consensus was not reached, then a third rater (JR) was consulted.

### Data Collection Process and Data Items

The following data was extracted from each study: i) reference details, ii) year of publication, iii) animal breed, iv) animals’ age, v) intervention length, vi) the WHAT: what elements the EE intervention added (number of animal peers, type of objects, structural layers, and other tasks), vii) the HOW: how the elements were added and managed throughout the intervention (disposition of the elements, type and frequency of changes), and viii) the NEEDS: which animal’s needs and interests were targeted (feeding, nesting, sheltering, playing, voluntary physical exercising, and exploring).

The data was extracted and compiled in a custom-made excel spreadsheet. The WHAT was directly reported in all studies. For the HOW: the type and frequency of changes were directly reported; the disposition was described in some studies, and it was inferred in other studies from the picture of the EE intervention. With regards to the NEEDS, animals’ needs were identified—feeding, nesting, sheltering, playing, voluntary physical exercising, and exploring—and subsequently coded for each study. A study was coded as targeting an animal’s need as follows: i) feeding, when the description highlighted that eating and drinking objects or their position were changed throughout the intervention, or the eating and drinking objects were positioned in different parts of the cage (described or observed in the figure), or the other added objects were positioned in front of the eating and drinking objects (described or observed in the figure); ii) nesting, when nesting materials were explicitly stated in the intervention description; iii) sheltering/feeling safe, when objects for hiding were added (e.g., little houses) in different areas or their position changed (described or observed in the figure); iv) playing, when objects animals can play with were added; v) voluntary activity, when running wheels were added; vi) exploring, when a variety of objects were added.

### Synthesis of Results

This review mapped the characteristics of the EE protocols adopted in the literature, and a descriptive synthesis was provided. Further, a thematic analysis was conducted to find patterns in the EE protocol characteristics (types and modalities of enrichment) and derive the principles that guided the design of the EE interventions. Then, all the included studies were categorised within the identified patterns to ensure that the observed patterns occurred across the studies, and it was not an isolated observation.

## Results

**Fig. 1:**
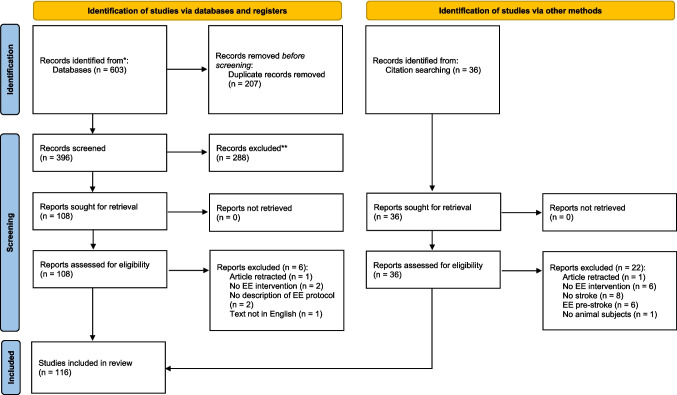
PRISMA flow diagram of the screening process

The search through the databases identified a total of 603 studies (Medline *n* = 161, PsycINFO *n* = 242, and Web of Science *n* = 200). After duplicate removal, the title and abstract of 396 studies were screened, and 288 were excluded. The full texts of 108 studies were screened and 102 studies met the inclusion criteria. An additional 14 studies were included from the reference list screening. A total of 116 studies were included in the review (Fig. 1).

### Description of Study Characteristics

A detailed description of the animals’ characteristics, what was enriched, how it was enriched, and the animals’ needs considered in the intervention is presented in Table 1.

**Table 1:** Characteristics of the included studies, including what was enriched, how it was enriched, and the needs and interests considered

				What				How			Needs And Interests					
Reference	Animal model	Animal age (weeks)	Intervention length (weeks)	Peers	Objects	Layers	Other	Disposition	Changes type	Changes per week	Feeding	Nesting	Sheltering	Playing	Voluntary activity	Exploring
Ohlsson & Johansson, 1995 [40]	Spontaneously hypertensive rats (SHR)	13	4	12	Chain, swing, swing board, and wooden blocks	2			Objects	1				x		x
Grabowski et al., 1995 [51]	SHR		13	12	Boards, chain, swing board, blocks	3			Objects, rearrangement	1				x		x
Johansson, 1996 [60]	SHR	16	5	7	Boards, chain, swing, swing board, blocks	3			Objects	1				x		x
Johansson & Ohlsson, 1996 [52]	SHR	12	12	9	Boards, chain, swing, swing board	2			Objects	1				x		x
Puurunen et al., 1997 [61]	Wistar rats		4	6—7	Tunnels, shelves, running wheel, balls, jars, objects	2			Objects	1			x	x	x	x
Mattsson et al., 1997 [62]	SHR		20		Boards, chain, swing board, blocks	2			Objects, rearrangement	2				x		x
Dahlqvist et al., 1999 [43]	SHR	10.5	7		Boards, inclined boards, chain, swing board, wooden blocks	3			Objects, rearrangement	1				x		x
Briones et al., 2000 [39]	Wistar rats	10	1.5		Ladders, swing, bells, woodblocks, running wheel, mirror, tunnels, cave, striped cardboard	1			Objects	7			x	x	x	x
Zhao et al., 2000 [63]	SHR	12	4	8—10	Boards, chain, swing board, blocks, balls	2			Objects, rearrangement	3				x		x
Puurunen et al., 2001 [64]	Wistar rats		4	6	Tunnels, shelves, running wheel, balls, jars, objects	2			Objects	3			x	x	x	x
Puurunen et al., 2001 [65]	Wistar rats		1.5	4—6	Tunnels, shelves, running wheel, balls, jars, objects	2			Objects	3			x	x	x	x
Zhao et al., 2001 [66]	SHR		1.5		Boards, various items	2								x		x
Biernaskie & Corbett, 2001 [67]	Sprague–Dawley rats		9	4—5	Shelves, plastic tubing, ladders, rope	2	Rehab-specific grasping	Intentional	Objects, rearrangement	2	x		x	x		x
Johansson, 2002 [50]	SHR	12		8	Boards, chain, swing board, blocks, balls	3			Objects, rearrangement	3				x		x
Komitova et al., 2002 [68]	SHR	24	5	12	Boards, chains, swings, blocks	1			Rearrangement	2				x		x
Risedal et al., 2002 [44]	SHR	14.5	4	8	Boards, chain, swing, swing board	2			Objects, rearrangement	2				x		x
Belayev et al., 2003 [69]	Wistar rats	21	8	6	Ramps, toys, running wheel, hammock, tunnels, branches	3			Objects, rearrangement	1			x	x	x	x
Xerri et al., 2003 [70]	Long-Evans rats	12	3	10	Mobile and immobile objects	1			Objects	7				x		x
Dahlqvist et al., 2003 [42]	SHR	14.5	4	8	Boards, chain, swing, blocks				Objects, rearrangement	2				x		x
Dahlqvist et al., 2004 [41]	Sprague–Dawley rats	7.5	5	12	Boards, ladders, tunnels, running wheel, chain, swing	2			Objects, rearrangement	7			x	x	x	x
Gobbo & O'Mara, 2004 [71]	Wistar rats		4	4	Running wheels, tunnels, toys	1			Objects	1			x	x	x	x
Komitova et al., 2005 [72]	SHR	24	5	9—10	Boards, chains, swings, blocks	1			Rearrangement	2				x		x
Komitova et al., 2005 [73]	SHR	24	1	10	Boards, chains, swings, blocks	1			Rearrangement	2				x		x
Nygren & Wieloch, 2005 [74]	C57BL/6 mice	10	4	10—15	Tubes, ropes, chains, ladders, ramps, platforms, running wheel, toys	2 +			Objects	3			x	x	x	x
Rönnbäck et al., 2005 [45]	Sprague–Dawley rats	7.5	4	8—10	Boards, ladders, tunnels, running wheel, chain, swing	2			Objects, rearrangement	7			x	x	x	x
Komitova et al., 2006 [75]	SHR	24	5	12	Boards, chains, swings, blocks	1			Rearrangement	2				x		x
Matsumori et al., 2006 [76]	Sprague–Dawley rats	10	8	10	Running wheel, labyrinth, bedding, ladder, house, chains, hammock, blocks, bones	2	Water-maze training		Rearrangement	1			x	x	x	x
Nygren et al., 2006 [77]	C57BL/6 mice	9.5	4	2—3	Toys, ramps, platforms	2 +								x		x
Sonninen et al., 2006 [78]	Wistar rats	12	3.5	4	Ladders, shelves, running wheel, balls, jars, objects	2			Objects	3				x	x	x
Dhanushkodi et al., 2007 [79]	Wistar rats	8	1.5	8—10	Tunnels, platforms, sandboxes, balls, rattles, sawdust, objects	3			Objects	7			x	x		x
Hicks et al., 2007 [80]	Sprague–Dawley rats		4	6	Tubes, beams, shelves, rope, ladder, running wheel	1			Objects, rearrangement	1			x	x	x	x
Saucier et al., 2007 [29]	Wistar rats		8.5	6	Balconies, ramps, balls, toys, chain, tubes	3 +		Intentional			x		x	x		x
Buchhold et al., 2007 [81]	Sprague–Dawley rats	12 and 80	4	8	Running wheel, catwalk, playing toys, hiding tunnel	1							x	x	x	x
Hicks et al., 2008 [82]	Sprague–Dawley rats	12	12	8	Tubes, beams, shelves, rope, ladders, running wheel	1			Rearrangement	1			x	x	x	x
Plane et al., 2008 [83]	Sprague–Dawley rats		7.5		Tunnels, ropes, wire mesh, chew toys, hidden snacks, toys	1			Rearrangement	7	x		x	x		x
Wang et al., 2008 [84]	Sprague–Dawley rats	10	4	5—6	Running wheel, labyrinth, bedding equipment, ladder, houses, chains, hammock, blocks, nylon bones	2			Rearrangement	1			x	x	x	x
Rickhag et al., 2008 [47]	Wistar rats		1.5	12	Chain, swing, swing board, wooden blocks	2			Objects	1				x		x
Hicks et al., 2009 [85]	Wistar rats			8—9	Toys, ladders, tubes, tunnels, shelves	1			Objects	1			x	x		x
Knieling et al., 2009 [86]	Wistar rats		4	7	Tubes, seesaws, running wheel				Objects	7			x	x	x	x
Ruscher et al., 2009 [48]	Wistar rats		1	4—7	Platforms, grids, pipes, ropes	2 +							x	x		x
Söderström et al., 2009 [87]	Female Wistar rats	6.5	6	15	Boards, ladders, tunnels, swings, chains	2 +			Objects, rearrangement	7			x	x		x
Xu et al., 2009 [26]	Sprague–Dawley rats		2	10	Toys, blocks, running wheels, tunnels, ladders, castles, swing	1	Open field for 10 min daily		Objects	7			x	x	x	x
Hirata et al., 2011 [49]	Sprague–Dawley rats	9	4	4—6	Running wheel, tunnel, balls, logs, rings	1			Rearrangement	2			x	x	x	x
Inacio et al., 2011 [88]	SHR	17	0.5			2 +										
Shono et al., 2011 [89]	Sprague–Dawley rats	9	4	4—6	Toys, running wheel, tunnel	1			Rearrangement	2	x		x	x	x	x
Zai et al., 2011 [90]	Sprague–Dawley rats		1	4—8	Ladders, platform, tunnels, toys, treats	1			Objects	7	x		x	x		x
Greifzu et al., 2014 [35]	C57BL/6 J mice	12 to 28			Running wheel, ladder, tunnel, maze	1		Intentional	Maze reconfiguration	3	x			x	x	x
Madinier et al., 2014 [46]	Sprague–Dawley rats	8	9	7	Beams, chains, tubes, ladders	2 +			Rearrangement	2			x	x		x
Quattromani et al., 2014 [91]	TLR2 + / − mice	12	2	5	Tunnels, houses, slides, running wheels	2 +			Rearrangement	3			x	x	x	x
Yu et al., 2014 [92]	Sprague–Dawley rats		3	8—12	Platforms, tubes, tunnels, chains, boxes	1			Animal substitution	2			x	x		x
Kuptsova et al., 2015 [93]	Wistar Han rats		4	8—9	Toys, ladders, tubes, tunnels, shelves	1			Objects	2			x	x		x
Wadowska et al., 2015 [27]	Wistar rats	12	2	22	Toys, blocks, running wheels, tunnels, ladders, castles, swing	1	Open field for 30 min daily		Objects	7			x	x	x	x
Cho et al., 2016 [94]	Mice	6	8	12 – 15	Tunnels, shelters, toys, running wheels	1		Intentional			x		x	x	x	x
Jiang et al., 2016 [95]	Sprague–Dawley rats		2	12	Climbing platforms, tubes, tunnels, chains, boxes	3		Intentional	Animal substitution	2	x		x	x	x	x
Kim et al., 2016 [96]	Sprague–Dawley rats	17	4	10	Tubes, running wheel, ladder, ball, rope	1			Objects, rearrangement	2			x	x	x	x
Su et al., 2016 [97]	Wistar rats		2	12	Climbing platforms, tubes, tunnels, chains, boxes	1			Objects	2			x	x		x
Wang et al., 2016 [98]	Kunming mice	8	2		Toys, running wheel	1			Objects	2				x	x	x
Ahmadalipour et al., 2017 [99]	Wistar rats	3	5.5	12	Running wheel, raised platform, tunnels, steel chains, balls, dolls	2			Objects	1			x	x	x	x
Chen et al., 2017 [100]	C57BL/6 mice	9	10	10	Tunnels, shelters, toys, running wheels	1							x	x	x	x
Chen et al., 2017 [101]	Sprague–Dawley rats		2	4	Toys, tubes, climbing ladders, running wheel	1							x	x	x	x
Chen et al., 2017 [102]	Sprague–Dawley rats		2	6—8	Climbing ladders, chains, tubes, tunnels, blocks, running wheel	1			Objects	7			x	x	x	x
Zhang et al., 2017 [103]	Sprague–Dawley rats		2	10—12	Ladders, chains, tubes, tunnels, blocks	1			Objects	7	x		x	x		x
Goncalves et al., 2018 [104]	C57Bl/6 mice	3	5	7	Running wheel, miniature house, toys	1			Objects	1			x	x	x	x
Jeffers & Corbett, 2018 [105]	Sprague–Dawley rats		4	5—6	Toys, beams, tools for climbing, food pellets	3	Rehab-specific grasping	Intentional			x		x	x		x
Wu et al., 2018 [106]	C57BL/6 mice	9	10	12—15	Tunnels, shelters, toys, running wheels	1							x	x	x	x
Zhang et al., 2018 [107]	C57BL/6 mice	9	10	5 – 8	Tunnels, shelters, toys, running wheels	1							x	x	x	x
Qian et al., 2018 [30]	Sprague–Dawley rats	9	2	7	Tunnels, stainless-steel bottoms, toys, ropes, shelves, balls, bells, houses	3		Intentional	Objects, rearrangement	2	x		x	x		x
Li et al., 2019 [108]	Sprague–Dawley rats	8	4	8—10	Ladders, chains, tubes, tunnels, boxes	1			Objects	3			x	x		x
Schuch et al., 2019 [36]	Sprague–Dawley rats		5	6 – 7	Running wheel, ladders, beams, ramps, containers, food reward, toys	3 +		Intentional	Objects, rearrangement	1	x		x	x	x	x
Tang et al., 2019 [109]	Sprague–Dawley rats		5	8—10	Running wheels, ladders, nest boxes, hammock, blocks, tunnels	1		Intentional	Objects	3	x	x	x	x	x	x
Wahl et al., 2019 [37]	Female Long-Evans rats	14	5	14 – 23	Ladders, running wheel, beams, food reward, training stations, vertical rope, houses, platform	3	Rehab-specific grasping	Intentional			x		x	x	x	x
Wang et al., 2019 [22]	C57BL/6 mice		2.5	10	Climbing ladders, tubes, tunnels, running wheels, boxes	1		Intentional	Objects	2			x	x	x	x
Wang et al., 2019 [23]	C57BL/6 mice	10	3.5	10	Climbing ladders, tubes, tunnels, running wheels, boxes	1		Intentional	Objects	2			x	x	x	x
Xie et al., 2019 [53]	Sprague–Dawley rats	7.5	4	6	Boxes, chains, barrels, ladders, toys, running wheels	1			Objects	7				x	x	x
Zhan et al., 2019 [110]	Sprague–Dawley rats	8	4	10 – 12	Tubes, tunnels, ladders, balance beam	1			Objects	3			x	x		x
Zhang et al., 2019[111]	C57BL/6 J mice	10	3		Chains, runners, ladders, pipelines, boxes	1			Objects	7			x	x		x
Hase et al., 2019 [112]	Sprague–Dawley rats	9	12		Running wheels, hanging chains, igloos, paper tunnel	1							x	x	x	x
Zhan et al., 2020 [31]	Sprague–Dawley rats	8	4	12	Running wheel, cabins, tunnels, swing boards, ladders, balance beams, multi food delivery, palatable food	1, 2, and 3 layers	Rehab-specific grasping	Intentional	Layers, objects, feeding route	7	x		x	x	x	x
de Boer et al., 2020 [113]	C57BL/6 J mice		5	8—10	Ladder, running wheel, chains, objects, tunnels	1	Toys added from day 2						x	x	x	x
Li et al., 2020 [114]	Sprague–Dawley rats	8	4	8—12	Ladders, chains, tubes, tunnels, boxes	1			Objects	3			x	x		x
Lin et al., 2020 [115]	HDAC2flox/flox and C57BL/6 mice	6.5	4.5	6	Running wheels, tunnels, igloos, huts, retreats, toys	1			Objects	3			x	x	x	x
Shen et al., 2020 [116]	Sprague–Dawley rats	10	2	12	Platforms, ladders, baffles, boards, swings, ropes, cassettes, tunnels, animal statues, balls, acoustic luminescent and odorous objects, building blocks, shapes, running wheels, edible foam	2		Intentional	Rearrangement	1	x		x	x	x	x
Wang et al., 2020 [117]	C57BL/6 mice	10	3	12	Slopes, ladders, platforms, tunnels, boxes, blocks, swings, running wheels	2		Intentional	Objects, rearrangement	2			x	x	x	x
Xie et al., 2020 [54]	Sprague–Dawley rats		4	6	Boxes, chains, barrels, ladders, running wheels	1			Objects	7				x	x	x
Yu et al., 2020 [25]	C57BL/6 mice	10	2	14	Climbing ladders, sports wheels, tubes, toys, tunnels, running wheels, huts, decorations	1		Intentional		2	x		x	x	x	x
Yuan et al., 2020 [118]	Sprague–Dawley rats		4	6—8	Ladders, platform, toys, tunnels	1			Objects	3			x	x		x
Deng et al., 2021 [32]	Sprague–Dawley rats	9.5	4	10 – 12	Toys, ladders, tunnels, blocks, tubes, platforms, chains	3	Rehab-specific grasping	Intentional	Objects	3	x		x	x		x
Ergen et al., 2021 [119]	Female Sprague–Dawley rats		2	7	Stairs, platforms, objects	2			Objects	3				x		x
Ip et al., 2021 [120]	Sprague–Dawley rats	10	3	10	Running wheel, labyrinth, bedding, ladder, house, chains, hammock, blocks, nylon bones	2			Rearrangement	1			x	x	x	x
Lin et al., 2021 [121]	C57BL6/J mice		4.5	6	Running wheels, tunnels, igloos, huts, retreats, toys	1			Objects	3			x	x	x	x
Liu et al., 2021 [122]	Sprague–Dawley rats	6.5	3.5	6—10	Ladders, platforms, swings, balls, blocks, tunnels, running wheel	2			Objects, rearrangement	3			x	x	x	x
Tan et al., 2021 [38]	Sprague–Dawley rats	7.5	1	8	Ladders, running wheel, shelter, bridge, toys, fragrant balls, hanging chains	2	Soft music and lighting	Intentional	Objects, rearrangement	7	x		x	x	x	x
Zhang et al., 2021 [28]	Sprague–Dawley rats		2	6—10	Bedding materials, balls, blocks, tunnels, ladders, running wheel	2			Objects, rearrangement	3			x	x	x	x
Zhu et al., 2021 [123]	C57BL/6 J mice	8	2	15	Houses, running wheels, ladders, tunnels, toys	2		Intentional	Objects, rearrangement	2	x		x	x	x	x
Zhang et al., 2021 [124]	Sprague–Dawley rats	10	4	6—8	Climbing ladder, chain, tubes, tunnel, screen cover with colour block, running wheel	1			Objects	7			x	x	x	x
Farajdokht et al., 2022 [125]	Balb/c mice		2		Running wheel, toys, balls, tunnels, ramps	1			Rearrangement	1			x	x	x	x
Gresita et al., 2022 [126]	Sprague–Dawley rats	18 and 78	4	10	Running wheel, catwalk, toys, tunnels	1							x	x	x	x
Guo et al., 2022 [24]	Sprague–Dawley rats	7	4	7	Ladder, pipes, tunnels, running wheels, boxes	1							x	x	x	x
Zhou et al., 2022 [127]	C57BL/6 mice	8	4		Spiral staircases, tunnels, running wheel, turntables, swings, blocks, houses, bell sound, colour light stimulation	3		Intentional	unspecified	2	x		x	x	x	x
Zhang et al., 2022 [128]	C57BL/6 mice	10	7	11	Fun room, running wheel, tubes, sports room, blocks	1			Objects, rearrangement	2			x	x	x	x
Chen et al., 2023 [129]	Sprague–Dawley rats		3	10 – 12	Climbing ladders, tunnels, shelters, toys, tubes, running wheels	1							x	x	x	x
Chen et al., 2023 [130]	Sprague–Dawley rats	8	3	10 – 12	Balls, tunnels, wooden blocks, running wheel	1		Intentional			x		x	x	x	x
Jia et al., 2023 [131]	C57BL/6 mice	10	4	6—8	Running wheels, igloos with saucer wheels, tubing, toys	1			Objects, rearrangement	7			x	x	x	x
Lee et al., 2023 [132]	C57BL/6 mice		2	12 – 15	Tunnels, shelters, toys, running wheels	1							x	x	x	x
Liu et al., 2023 [133]	Sprague–Dawley rats	6.5	3	6	Stairways, stages, swing boards, tunnels, running wheels	2		Intentional		2	x		x	x	x	x
Luo et al., 2023 [134]	Sprague–Dawley rats	6.5	3	8—12	Running wheels, platforms, ladders, tunnels	2		Intentional	Rearrangement	2			x	x	x	x
Shi et al., 2023 [135]	C57BL/6 mice	12	3		Houses, ladders, tubes, running wheels	1			Objects	2			x	x	x	x
Woitke et al., 2023 [136]	C57Bl/6 J mice	12	7	6—7	Tubes, running wheels, houses	1			rearrangement	7			x	x	x	x
Yan et al., 2023 [137]	Sprague–Dawley rats	76	4		Running wheel, catwalk, toys, tunnel	1							x	x	x	x
Zhang et al., 2023 [138]	C57BL/6 mice	36	6	6	Tunnels, climbing frame, platforms, idling shelters, house, running wheel	2			Objects, rearrangement	2			x	x	x	x
Guo & Bi, 2024 [139]	Sprague–Dawley rats	7	3.5	10	Boxes, chains, balls, tunnels, running wheels	1			Objects	2			x	x	x	x
Han et al., 2024 [140]	Sprague–Dawley rats	8	4		Nesting materials, running wheels, ladders, boards, swings, balls, blocks	3		Intentional	Objects, rearrangement	2		x	x	x	x	x
Huang et al., 2024 [141]	C57BL/6 J mice	7	1	8—10	Running wheel, ladders, tubes, boxes	3		Intentional	Objects, rearrangement	7	x		x	x	x	x
Lu et al., 2024 [33]	Sprague–Dawley rats	8	4	10 – 12	1) toys, running wheels; 2) balance beams, swing boards, ladders, tunnels; 3) floating cabins, balance beams, ladders, food pellets	3	Rehab-specific grasping	Intentional	Objects, rearrangement	7	x		x	x	x	x
Lu et al., 2024 [34]	Sprague–Dawley rats	8	4	10—12	1) toys, running wheels; 2) balance beams, swing boards, ladders, tunnels; 3) floating cabins, balance beams, ladders, food pellets	3	Rehab-specific grasping	Intentional	Objects, rearrangement	7	x		x	x	x	x
Martinez-Torres et al., 2024 [142]	Sprague–Dawley rats		1.5	5	Stairs, chewable toys, pipes, climbing bridges, running wheels, tape with Nutella® and different smells	1			Objects, rearrangement	3	x		x	x	x	x

#### Animals’ Characteristics and Intervention Length

Seventy-six percent of the studies used rats (spontaneously hypertensive, Wistar, Sprague–Dawley, Long-Evans, and Wistar Han), and the remaining 24% of the studies used mice (C57BL/6, C57BL/6 J, Balb/c, HDAC2flox/flox, Kunming, and TLR2 + / −). The spontaneously hypertensive rats were the only animals to present co-morbidities, as hypertension is a known co-morbidity in stroke. All the animals used in the other studies were without co-morbidities. All studies used male animals, except for Ergen et al., 2021 and Soderstrom et al., 2009 that used female animals. The animals’ age ranged from 3 to 80 weeks (mean = 13.7, SD = 13.8), meaning that the animals were on average young adults, given that mice and rats are considered to enter adulthood at approximately 6-to-12 weeks of age when they are sexually mature [20, 21].

The length of the interventions ranged from half week to 20 weeks (mean = 4.4, SD = 2.9).

#### What Was Enriched

**Fig. 2:**
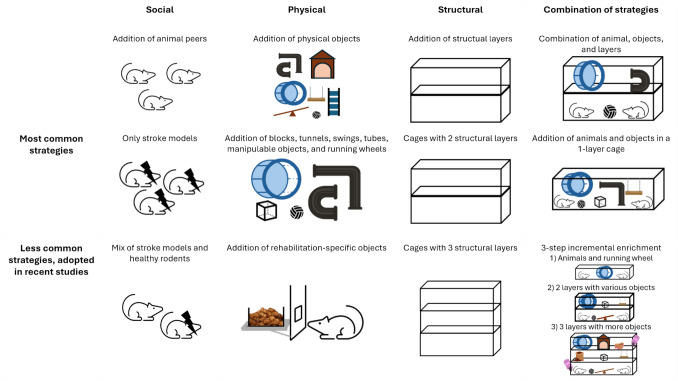
Visualization of social, physical, and structural enrichment strategies, with the most and less common approaches

Eighty-seven percent of the studies added animal peers, with an average number of 8.5 animals per cage; 99% added various objects; 47% added structural layers to the cage. The animals added to the cage were in most cases post-stroke models, except for [22–28] that mixed healthy mice and post-stroke models. The objects added to the cage were various, with the most common being blocks, tunnels, swings, tubes, manipulable objects, and running wheels. The structural layers added to the cage created different levels which animals could explore, with the most common being 2-layer cages (Fig. 2).

These strategies can be categorised in three types of enrichment: social (animal peers), physical (objects), and structural (cage layers). Eighty-seven percent of studies implemented social, 99% physical, and 47% structural enrichment.

#### How It Was Enriched

Only 9% of studies provided a description of how the objects or at least some of them were arranged within the cage and 26% provided a picture of the enriched cage. All the descriptions indicated where food and/or water were positioned within the cage [29–34] (e.g., food pellets were placed on the top floor [30]) or how other objects were positioned in reference to food and/or water [35–38] (e.g., the maze separates food and water compartments [35]), except for Briones, Therrien [39] which indicated a random disposition of the enriching elements. Our observation of the pictures showed two main trends: i) in 1-level cages, objects were scattered around, and ii) in multilevel cages, objects related to the different animals’ functions were positioned in the different cage levels (e.g., running wheel and manipulable objects at the bottom level, sheltering at the middle, and food at the top level).

 Eighty percent of the studies made weekly changes throughout the intervention, with 46% of studies changing the objects, 19% of studies rearranging the position of the objects, 31% of studies combining these two strategies, and the remaining 4% rotating animals or changing the layers. The frequency of the changes spanned from 1 to 7 changes per week with an average of 3.3 changes per week.

All this can be categorised in two modalities of enrichment: the addition of new elements and the manipulation of existing elements. All studies added new elements at the beginning of the intervention to set up the enriched cage, and 80% of them manipulated existing elements throughout the intervention.

#### Animals’ Needs Considered in the Intervention

Ninety-eight percent of the studies targeted the animals’ needs and interests to play and explore, 77% their need to shelter and feel safe, 64% their need to perform voluntary physical activity (on a running wheel), 23% their need to eat and drink, and 2% their needs to nest. Considering that these needs were inferred from the EE description and picture, and the descriptions were limited in the earlier studies, the percentage could be higher, especially for the feeding need.

### Emerging Themes and Principles

#### Principles Guiding the Enrichment

After the initial reading and reflection on the EE characteristics of the included studies, and in combination with existing literature [3, 13, 15, 16], six thematic principles were identified: complexity (spatial and social), variety, novelty, targeting needs, scaffolding, and integration of rehabilitation tasks. These principles and their implementation in each study were defined and coded as follows: i) complexity, when different types of objects and structural layers (spatial complexity) and multiple animals (social complexity) were included to create a complex rich environment, ii) variety, when multiple objects were added to provide a variety of stimuli, iii) novelty, when the added elements or their position were changed throughout the intervention to constantly provide novel stimuli, iv) targeting needs, when the intervention intentionally targeted at least one of the animal’s needs, v) scaffolding, when the EE intervention was implemented progressively and incrementally, and vi) integration of rehabilitation activities, when tasks were intentionally implemented to stimulate the activity of the body functions affected by the stroke (e.g., grasping equipment that forced animal to use their affected limb to grasp food pellet).

All studies combined complexity and variety through having multiple objects in the cage and different structural layers that provided richness in stimuli. Eighty percent of the studies implemented novelty through the substitution of objects (and animals in some instances) and re-organization of the spatial disposition of the enriching elements throughout the intervention. Sixteen percent of studies explicitly described the integration of rehabilitation tasks within the enrichment intervention, such as equipment that forced animals to use their affected limbs to grasp and eat food pellet. This percentage (16%) is likely to be higher, as the addition and substitution of objects require animals to use their affected limb to conduct daily activities. Lastly, three percent of studies scaffolded their enrichment intervention through incremental addition of social, physical, and structural elements.

All studies implemented enrichment within the animals’ daily life. The enriching elements were used by animals to carry out their daily life activities, such as structural layers and ladders to access food, added tubes to be used as shelter, objects that stimulate exploration, and tubes for going from one place to the other of the cage to access elements fundamental to satisfy the animals’ needs.

#### Historical Trend

The first article using an EE intervention on post-stroke animal models was published in 1995 [40] and since then there has been a steady increase in the number of publications. The description and most likely understanding of EE protocols became more thorough throughout the years. Early publications provided a limited description of their EE protocol, highlighting which elements were initially added to set up the cage and how they were subsequently manipulated. This description and the depth of animals’ needs targeted improved throughout the years. The animals’ needs of playing and exploring were mostly targeted initially, which then expanded to physical activity, sheltering, and feeding. In the past decade, studies implemented comprehensive and complex EE protocols, integrating rehabilitation-specific stimuli and scaffolding the enrichment strategy to “guide” the animals’ interaction with the enriched elements.

#### Lab-Led Enrichment

Most of the EE protocols implemented in the included studies were derived from previously published EE protocols, in most cases conducted in the same lab. For instance, a series of studies from the same lab or partnering labs [41–52] based their intervention on the protocol published by Ohlsson and Johansson [40]. None of the included studies explicitly based their EE protocol on theories or frameworks that consider the complex interaction of an animal with their environment and how an environment can be modified to stimulate certain behaviour of an animal. In most, if not all, the included studies the goal of their EE intervention (and their rationale) was to promote experience-dependent plasticity, but from a design perspective, it can be said that enrichment protocols have been mostly intuition-based and followed existing lab protocols.

#### How the Level of Enrichment Has Been Assessed

Four studies assessed the level of enrichment through the measurement of animals’ interaction with and exploration of the enriching elements and their physical activity in the different areas of the cage. To do so, 3 methods were used: i) a radio-frequency identification system that comprises a transponder attached to each individual animal and antennas scattered around the cage [36, 37], ii) video observation from an infrared camera that allowed the recording of animals’ activity throughout the day [37], and iii) an automated tracking system that uses an infrared camera [53, 54]. While these studies averaged results across animals to compare the different experimental conditions, these tracking methods can be used to measure the amount each animal interacted with the presented EE stimuli. These techniques can also be used to identify the right timing for manipulating the enriching elements and maintain a high level of stimulation, e.g., increase novelty when animals’ activity and interaction decrease.

## Discussion

This study provides a detailed overview of the EE protocols published to date in the preclinical post-stroke literature, collating data from 116 experimental studies and showing a series of principles and processes that underpinned the design of EE interventions with animal models of stroke. These findings are summarized and discussed to answer the research question of this study: what constitutes EE and its guiding principles in preclinical post-stroke. This is then discussed as a foundational starting point for the translation to clinical post-stroke.

### Main Findings

The main finding from this review was the breadth of EE protocols and interventions adopted in the included studies, which shared many commonalities as well as some interesting differences. The core of the EE interventions was comprised of an initial set up of the enriched cage, with objects, animal peers, and in some cases structural layers, and the subsequent manipulation of the existing elements throughout the intervention. The addition and manipulation of the enriching elements appeared not to be random but geared towards targeting animals’ needs and stimulating them to constantly engage with the environment and perform cognitive and motor activities. Placing a variety of objects in the cage and changing them or their position regularly, placing little houses and cubes, and placing feeding-water tanks at different levels of the cage were the most used strategies.

Our critical reflection of these EE protocols and strategies unveiled the overarching principles that guided the EE design. *Complexity*, *variety*, *novelty* of the environment, and *targeting needs* of the animals were the most used principles, as highlighted in previous literature [14, 15]. The animals’ needs considered in the analysed EE protocols were inferred to exploring, playing, performing physical activity, feeding, sheltering, and nesting. The animals’ needs were targeted to encourage animals to engage in motor, cognitive, and sensory activities to explore the environment. Other principles emerged—scaffolding and integration of rehabilitation tasks—in a small subset of the included studies (all published in the past 5 years). EE was embedded within the animals’ life, allowing for animals to carry out their daily activities. Collectively, this shows that EE is not the simple set up of an environment with the random addition of objects, but it is a complex process that requires careful consideration of the animals’ needs and behaviour and regular adjustments of the enriched environment to ensure continuous stimulation to the animals. These principles combined reveal that the core of an EE intervention is the interactions of an animal with their environment, the coupling of which cascades into enhanced motor, cognitive, and exploratory activities promoting neuroplasticity mechanisms.

### What Constitutes EE in Preclinical Post-stroke Studies

EE is a strategic modification of the environment that creates frequent opportunities and invitations for a variety of physical, cognitive, and exploratory activities, encouraging extensive use of the body functions affected by the stroke. These opportunities and invitations for active behaviour are created through the addition of new elements (at the beginning of the intervention) and frequent manipulation of existing elements (throughout the intervention) across the physical, social, and structural environment. Enrichment is embedded within the animals’ daily environment, comprehensively affecting their needs and functions, inviting them to frequently interact throughout the day with the elements of their surrounding environment. Interactions with the environment that allow animals to carry out their daily basic needs, such as eating, sheltering, and exploring. In short, it is the animals’ daily environment that is enriched.

We intentionally use both the term “opportunities” and “invitations” to capture the saliency that different environmental modifications can have on animals. Some environmental interventions provide animal with an opportunity for movement and cognitive activities. For instance, a running wheel allows animals to perform voluntary physical activity. They engage with the running wheel only if they want to. Other environmental modifications were not simple opportunities for animals that they can use if and when they wanted, but the modifications were highly salient invitations to engage with the enriching elements to conduct daily activities. For instance, ladders were not randomly placed in the cage offering a mere opportunity for climbing, which animals decided whether to engage with or not. Instead, in most cases, the ladders were connected to a higher level that contained important elements for the animal (e.g., food and shelter), in turn inviting them to use it—if they wanted to reach the food they had to climb the ladder. It follows that the invitations must be tailored to the needs and relevant for the individual inhabiting the EE to promote their engagement with the environment. An animal will engage with the ladder if the ladder is scaled to his body size (i.e., the animal has the capacity to climb it) and if the food is appetizing to it. Further, given the variety of modifications and frequency of enrichment changes, invitations are tailored towards the continuous performance of a variety of motor-perceptual-cognitive-exploratory activities.

In sum, EE for post-stroke is a strategy that frequently modifies the animals’ daily environment to create a richness of spatial, structural, and/or social invitations and opportunities to engage in a variety of daily-related motor-cognitive-social exploratory activities that are relevant and tailored to the inhabiting individual and involve activation of the affected body function(s). This complex process requires careful consideration of the animal-environment relationship and regular adjustments of the enrichment to ensure continuous occasions and invitations to the animals for cognitive-motor-exploratory interactions.

This definition of what constitutes EE adds key concepts to the most common EE definitions (e.g., see [2, 3, 10, 11]). EE provides invitations and not stimulations; an environment is enriched when it is relevant to the animal and fits their characteristics and needs; enriching is a continuously evolving process, requiring frequent adjustments, at the interface between environmental features and the characteristics of the inhabiting individual; as highlighted in McDonald, Hayward [3] definition, EE for post-stroke should provide invitations to engage in extensive activities with the affected body function(s).

### What Principles Can Guide an EE Intervention

The strategies of EE were guided by existing and new principles. *Complexity* provides animals with an abundance of rich stimuli that encourage both motor and cognitive exploratory activities. Complexity in most cases implies an environment that requires the animal to use their affected limbs to find solutions to functional challenges related to their needs, such as climbing a ladder to reach food. Previous research has mostly considered spatial complexity, in terms of the number and type of elements (animate and inanimate) added into a cage and their changes throughout an intervention (the higher the number of elements and changes the higher the complexity) [16, 55, 56], showing how complexity is instrumental in promoting neuroplasticity [16]. This view on complexity emphasizes its interconnectedness with variety and novelty, and it can be said that variety and novelty contribute to the complexity of an environment. *Variety* in the furnishings of the environment offers multiple solutions to tasks, promoting continuous invitations for exploration and adaptation of movement strategies. Previous research has shown that when novelty is combined with complexity, effects are enhanced, rendering novelty an important factor for promoting cognitive and behavioural improvements in mice [17, 57]. As movement exploration wanes, *novelty* is introduced by adding new stimuli to the environment, creating fresh movement opportunities and invitations to explore.

*Targeting the animal’s needs,* through manipulation of daily habits and routines, ensures that the environment is relevant to the animals and fosters continuous exploration. Throughout all these principles and, where possible, *rehabilitation activities* are seamlessly *integrated* to encourage the engagement of affected body parts during movement. Targeting the animals’ needs and integrating rehabilitation activities for the affected body functions are critical for promoting neuroplasticity, i.e., neuroplasticity is activity- and experience-dependent requiring extensive use of the affected body function [4]. Interestingly, some of the included studies implemented the principles of *scaffolding* to avoid overloading the animal and progressively expose them to an increase range of invitations.

These principles and their combination emphasize that enrichment is primarily a pedagogical intervention, made of strategic decisions to modify an environment with the aim of increasing motor, cognitive, and exploratory activity. The recipe for enrichment does not exist, and enrichment is not a black and white process, whereby an environment can be classified as either enriched or non-enriched. Instead, the studies included in this review showed that it is an evolving process that requires an intervening actor or agent to frequently observe the animals’ behaviour and their interaction with the environment and to make adjustment to maintain animals’ engagement with their surroundings. The principles discussed above can guide an investigator throughout this process.

### Considerations for the Translation to Clinical Post-stroke

The findings of this review show that enrichment is not the simple addition of objects to an impoverished environment. It is a comprehensive intervention that requires careful consideration of the characteristics and needs of the individual within the EE, how the environment can be modified to satisfy those needs, and what opportunities for cognitive and motor activities the enriched environment offers to the individuals inhabiting it. Unpacking our definition of what constitutes an EE for post-stroke in combination with principles identified in this review, we can start providing some stepping stones for the translation of EE to human clinical settings.

The first stepping stone for the clinical translation is the understanding that enrichment is an evolving pedagogical process aiming at creating invitations and opportunities for patients’ motor and cognitive exploratory behaviour. Invitations and opportunities for behaviour differ between lab animals and stroke patients, and a critical reflection on how these emerge in humans and how they depend on individual patient characteristics should lead the translation. Second, the principles identified in this review should be tailored and to the human context. What is the meaning of *complexity, novelty, variety, targeting needs, integrating rehabilitation activities, and scaffolding* for stroke patients, and how can these principles be implemented and manipulated across the different phases of the rehabilitation process (e.g., in the hospital and in the patient’s home)? Furthermore, what other principles from relevant disciplines (e.g., physiotherapy, neurology, neuropsychology, rehabilitation science, pedagogy and didactics) should be considered to create a comprehensive intervention that targets critical aspects of the rehabilitation process? Third, how can modifications be made in the patients’ daily environment in a way that is acceptable for them? How far can modifications go in changing patients’ routine? Fourth, enrichment is not a one-off intervention that necessarily requires the building of a new physical environment, but rather an evolving intervention that requires frequent manipulation of the environment to ensure it provides invitations for active and exploratory behaviour to patients. This study does not provide solutions to these issues, but the review of the animal literature gives a direction for key principles to be explored further in human EE studies. Establishing new clinical practices based on basic science findings is a complex and lengthy issue, and this study hopefully provides key concepts that may be a theoretical starting point for researchers and clinicians embarking on the journey of translating the EE paradigm to human stroke rehabilitation.

### Limitation of this Review and Avenues for Future Research

This review did not synthesize the effectiveness of different EE protocols on stroke-related outcomes. This is, however, an important aspect that future research should investigate, as it will elucidate the impact of different EE strategies and approaches on stroke recovery. Another critical avenue for future translational research is the systematic investigation and standardization of EE in non-human primates, which are increasingly used in translational stroke research [58, 59]. The EE guiding principles delineated in this review could be tested in non-human primate models of stroke to bridge the gap between rodent models and humans.

## Conclusion

This scoping review mapped the EE protocols published to date in animal models of post stroke, revealing the characteristics of the animals used in the experiments, what was enriched (physical, social, and structural aspects), how it was enriched (strategic set up of the cage and frequent manipulations), and the animals’ needs considered (feeding, nesting, sheltering, playing, voluntary physical exercising, and exploring). Further, this review identified principles that were common to most studies—complexity, novelty, variety, targeting needs—and principles that were implemented in more recent studies—integration of rehabilitation activities and scaffolding. All this led to the definition of what constitutes EE in preclinical post-stroke research: EE for post-stroke is a strategy that frequently modifies the animals’ daily environment to create a richness of spatial, structural, and/or social invitations and opportunities to engage in a variety of daily life-related motor-cognitive-social exploratory activities that are relevant and tailored to the inhabiting individual and that involve activation of the affected body function(s). This definition and the principles identified can serve as steppingstones for the translation of the paradigm to human stroke rehabilitation.

## Supplementary Information

Below is the link to the electronic supplementary material.

Supplementary file1 (DOCX 21 KB)

Supplementary file2 (DOCX 85 KB)

Supplementary file3 (DOCX 22 KB)

## Data Availability

No datasets were generated or analysed during the current study.
