# Supplementary material for: A Scoping Review of Preclinical Environmental Enrichment Protocols in Models of Poststroke to Set the Foundations for Translating the Paradigm to Clinical Settings
Source: Transl Stroke Res. 2025 Feb 6;16(5):1850–73. doi: 10.1007/s12975-025-01335-3 (PMC12391244; doi:10.1007/s12975-025-01335-3)
Supplement: Supplementary file 1 — Supplementary file1 (DOCX 21 KB) [file 12975_2025_1335_MOESM1_ESM.docx]

Table 1 Eligibility criteria

| **Inclusion criteria** | **Exclusion criteria** |
| --- | --- |
| Population: animal models of stroke  Studies that induced stroke through cerebral ischemia or haemorrhage were included | Studies that induced other brain injuries or modelled other disease were excluded |
| Intervention: Environmental Enrichment after the stroke event  Studies that conducted an EE intervention – modification of the cage environment to provide social, cognitive, and motor stimulation – post stroke were included | Studies that conducted an EE intervention pre-stroke only or other type of interventions were excluded |
| Peer-reviewed articles  Language: English | Other literature, and publications in languages other than English were excluded |
