# Supplementary material for: A Scoping Review of Preclinical Environmental Enrichment Protocols in Models of Poststroke to Set the Foundations for Translating the Paradigm to Clinical Settings
Source: Transl Stroke Res. 2025 Feb 6;16(5):1850–73. doi: 10.1007/s12975-025-01335-3 (PMC12391244; doi:10.1007/s12975-025-01335-3)
Supplement: Supplementary file 3 — Supplementary file3 (DOCX 22 KB) [file 12975_2025_1335_MOESM3_ESM.docx]

**A scoping review of preclinical Environmental Enrichment protocols in models of poststroke to set the foundations for translating the paradigm to clinical settings**

**Translational Stroke Research**

Luca Oppici^1*^, Guna Bērziņa^2,3^, Ann Marie Hestetun-Mandrup^4,5^, Marianne Løvstad^4,6^, Arve Opheim^4,7^, Matheus M. Pacheco^8^, Lena Rafsten^7,9^, Katharina S. Sunnerhagen^7^, PEER-HOMEcare consortium, James R. Rudd^1,10^

^1^ Department of Teacher Education and Outdoor Studies, Norwegian School of Sport Sciences, 0863 Oslo, Norway

^2^ Department of rehabilitation, Faculty of Health and Sport Sciences, Riga Stradiņš University, Riga, Latvia

^3^ Clinic of Rehabilitation, Riga East University Hospital, Riga, Latvia

^4^ Sunnaas Rehabilitation Hospital, 1450 Nesoddtangen, Norway

^5^ Department of Rehabilitation Science and Health Technology, Oslo Metropolitan University, Oslo, Norway

^6^ Department of Psychology, University of Oslo, Oslo, Norway

^7^ Institute of Neuroscience and Physiology, Dept of Clinical Neuroscience and Rehabilitation Medicine, Sahlgrenska Academy, University of Gothenburg, Gothenburg, Sweden.

^8^ CIFI2D, Faculty of Sport, University of Porto, Porto, Portugal

^9^ Department of Occupational Therapy and Physiotherapy, Sahlgrenska University Hospital, Gothenburg, Sweden

^10^ Department of Sport, Food and Natural Sciences, Faculty of Education, Arts and Sports, Western Norway University of Applied Sciences, 6856 Sogndal, Norway

^*^Corresponding author: Luca Oppici, lucao@nih.no

**Search string**

**MEDLINE**: (stroke or "cerebral infarct" or "cerebrovascular accident" or "CVA" or "brain ischemia" or "focal cortical ischemia" or "cortical infarct" or "cerebral ischemia" or "MCA occlusion" or "middle cerebral artery occlusion" or "brain hypoxia").af. and ("enriched environment" or "environmental enrichment" or "enriched housing").ti.

**Psycinfo**: (stroke or "cerebral infarct" or "cerebrovascular accident" or "CVA" or "brain ischemia" or "focal cortical ischemia" or "cortical infarct" or "cerebral ischemia" or "MCA occlusion" or "middle cerebral artery occlusion" or "brain hypoxia").af. and ("enriched environment" or "environmental enrichment" or "enriched housing").ti.

**Web of Science**: (TS=(stroke OR “cerebral infarct” OR “cerebrovascular accident” OR “CVA” OR “brain ischemia” OR “focal cortical ischemia” OR “cortical infarct” OR “cerebral ischemia” OR “MCA occlusion” OR “middle cerebral artery occlusion” OR “brain hypoxia”)) AND TI=("enriched environment" OR "environmental enrichment" OR "enriched housing")
